# Enhancing Patient Education on Safe Crushing of Hazardous Oral Medications for Patients With Swallowing Difficulties at Home

**DOI:** 10.7759/cureus.97975

**Published:** 2025-11-27

**Authors:** Faisal A Aleidi, Meshal M AlRayys, Mohammed M Almotairi, Naif A Alsamreen, Fahad A Alshammari, Maha M AlNakiyah, Renad A Alhaqbani, Mohammed A BinQasim, Hatoon AlRayes, Lamyaa Alsubaie, Ahmed AlRassan

**Affiliations:** 1 Pharmacy Services Administration, King Fahad Medical City, Riyadh, SAU; 2 Clinical Pharmacy, King Fahad Medical City, Riyadh, SAU; 3 Pharmacy, King Fahad Medical City, Riyadh, SAU; 4 Pharmacy, Princess Nourah Bint Abdulrahman University, Riyadh, SAU; 5 Electronic Medical Records Administration, King Fahad Medical City, Riyadh, SAU

**Keywords:** crush, hazard materials, home healthcare, oncology pediatrics, tablet splitting

## Abstract

Background

Crushing oral hazardous medications (HMs) is often necessary for patients with swallowing difficulties; however, this practice poses significant health risks to caregivers and patients due to exposure to toxic particles and potential alteration of drug properties. Despite existing guidelines, practical implementation in home settings remains limited, especially in the absence of clear, accessible instructions.

Methods

This interventional study was conducted at a tertiary care hospital in Saudi Arabia. A formulary review identified 26 oral hazardous medications suitable for safe crushing, based on evidence from regulatory sources, leaflet products, and published literature. An educational intervention was developed, including bilingual infographics and standardized medication instructions disseminated via the hospital's electronic medical record (EMR) and mobile application. Fifty participants who were prescribed hazardous oral drugs and required manipulation at home completed a pre- and post-intervention survey assessing knowledge and practices related to preparation, storage, disposal, and spill management.

Results

Survey findings revealed significant gaps in baseline knowledge: only 17 (34%) participants recognized hazardous medications, 13 (26%) understood associated risks, and 4 (8%) demonstrated appropriate spill management. Following the pharmacist-led intervention, all knowledge domains improved significantly (p < 0.001), with all 50 (100%) participants correctly identifying medication risks, preparation steps, safe disposal practices, and appropriate spill response. A validated list of 26 oral hazardous medications with evidence-based crushing instructions was also compiled to guide safe manipulation at home.

Conclusion

This study highlights the effectiveness of pharmacist-led counseling and bilingual educational tools in improving caregiver awareness and safety practices when handling hazardous oral medications. Integration of standardized instructions into electronic systems, along with targeted education, can significantly reduce exposure risks in home settings. These findings support broader adoption of structured interventions to ensure safe medication handling and improve patient outcomes.

## Introduction

Hazardous medications (HMs) are drugs that pose significant risks to individuals handling them, including healthcare providers, caregivers, and patients [1,2]. These drugs include categories such as antineoplastics, immunosuppressants, antivirals, hormonal agents, and other drugs with specific risks, such as warfarin and certain antibiotics, all of which require careful handling due to their potential health hazards [1]. For a drug to be classified as hazardous, the National Institute for Occupational Safety and Health (NIOSH) requires it to meet at least one of the following criteria: carcinogenicity, teratogenicity or developmental toxicity, reproductive toxicity in humans, genotoxicity, organ toxicity at low doses in humans or animals, or structural or toxicity characteristics similar to known hazardous drugs [1].

HMs are handled in diverse settings, including clinical environments and homes, where unique challenges arise. For instance, patients with swallowing difficulties, such as the elderly, pediatric populations, or those with certain medical conditions, may require medications to be altered to facilitate administration [3]. These alterations, such as crushing tablets or opening capsules, are often necessary, especially when there is no availability of liquid formulations or ready-to-use extemporaneous preparations for hazardous medications [4]. While this ensures easier medication intake, it also introduces risks of improper handling and exposure to hazardous particles, which can have serious consequences for healthcare providers and caregivers [5]. Activities such as crushing tablets or opening capsules can release hazardous particles or residues, increasing the likelihood of exposure [4,6]. Common routes of exposure include inhalation, skin contact, and accidental ingestion [5]. Such exposure has been linked to serious health effects, including DNA damage, increased cancer risk, organ toxicity, and reproductive harm, such as fertility impairment and risks to fetal development [7-9].

Furthermore, some hazardous medications may have limited stability once altered, potentially reducing their efficacy or safety, adding another layer of complexity to their preparation and administration [7,8]. Despite these risks, altering hazardous medications, such as crushing or opening tablets, is often unavoidable for patients with swallowing difficulties or when no suitable alternative formulations are available [9]. Therefore, minimizing exposure during the handling of these drugs is critical.

To reduce these risks, it is essential to follow established guidelines for safe handling. The Safe Handling of Hazardous Drugs Guideline Development Group (GDG) recommends personal protective equipment (PPE), such as gloves, gowns, and face masks, to prevent exposure in clinical settings. Crushing should be done in well-ventilated areas using containers such as plastic pouches to contain hazardous particles [10,11]. In home settings, where advanced PPE may not be available, simpler tools such as tissues, table covers, and gloves can help reduce exposure. Proper disposal of crushed medications and related waste is essential to avoid contamination [12]. Addressing these challenges requires targeted interventions.

The study first aims to develop a comprehensive list of oral medications that can be safely crushed at home, based on reliable scientific evidence, to serve as a practical reference for patients and caregivers in making safe and effective medication-related decisions. It also seeks to improve the safe handling of crushed oral HMs for patients with swallowing difficulties by educating caregivers on proper crushing techniques, the use of PPE, and appropriate storage and disposal practices in accordance with established safety guidelines. Additionally, the study aims to streamline pharmacy consultations by providing standardized, multilingual instructions through electronic health platforms, thereby reducing exposure risks and enhancing medication safety in home settings.

## Materials and methods

Study design and participants

This interventional study was conducted at King Fahad Medical City (KFMC), Riyadh, Saudi Arabia, between September 2024 and May 2025, with the aim of improving patient knowledge and safety practices related to hazardous oral medication handling. The study focused on patients who were prescribed oral hazardous medications requiring manipulation (e.g., crushing) at home. Patients were eligible for inclusion if they were prescribed a hazardous medication from the hospital formulary and received counseling through the hospital's electronic medical record (EMR) or mobile application. All participants received the intervention materials in both Arabic and English. Patients who declined counseling or failed to complete either the pre- or post-intervention surveys were excluded.

An extensive review was conducted to evaluate the formulary oral hazardous medications (HMs) available at our tertiary care hospital. A total of 70 oral HM items were initially identified. Of these, 45 items were excluded based on one or more of the following criteria: (1) modified-release formulations, (2) lack of evidence supporting safe crushing practices, and (3) potential alteration of pharmacokinetic properties upon crushing. The final list included 26 oral HM items with validated crushing instructions based on evidence from the British Oncology Pharmacy Association (BOPA) version 2.0 (2023) [13], UpToDate Lexidrug (2024) [14], AusDI (2024) [15], manufacturer leaflets, published reviews, and case reports. Recognizing the limited and occasionally conflicting data across these sources, we systematically documented the reference used for each medication according to the country of applicability. In cases of discrepancy, priority was given first to manufacturer data, followed by regulatory guidelines and published literature. This approach was designed to maximize transparency, scientific rigor, and reproducibility of the resulting list. The included medications were as follows: acitretin, bosentan, chlorambucil, crizotinib, dasatinib, enzalutamide, everolimus, exemestane, lenalidomide, lomustine, macitentan, megestrol, melphalan, misoprostol, nilotinib, paroxetine, phenoxybenzamine, propylthiouracil, rasagiline, riociguat, sorafenib, sunitinib, tamoxifen, tofacitinib, tretinoin, and warfarin.

Educational intervention

To minimize occupational exposure risks during home manipulation of oral hazardous drugs, an educational infographic was developed based on international safety recommendations. The content emphasized safe medication preparation, use of personal protective equipment (PPE), safe disposal practices, and response to accidental spills. The infographic was created in Arabic and English using simplified visuals and clear language to ensure accessibility for patients and caregivers.

Medication instructions were disseminated through the hospital's electronic medical record system (EPIC), whereas the educational infographic was delivered via the official IKFMC mobile application. Counseling also included recommendations on the use of household PPE and handling tools, guided by NIOSH (2023) [5], Oncology Nursing Society protocols [16], and Nationwide Children's Hospital [17]. The full infographic is available in the Appendices.

Survey instrument

A pre- and post-intervention survey instrument was developed by the principal investigator. The content validity of the questionnaire was established through expert review by two oncology pediatric clinical pharmacists, who assessed its clarity, relevance, and comprehensiveness. The survey was pilot-tested with five patients to ensure clarity and comprehension; these participants were later included in the final study sample. The validated survey was available in both Arabic and English and administered electronically. It consisted of six multiple-choice questions covering the following domains: basic knowledge of hazardous medications and associated risks, understanding of proper preparation techniques, storage practices, disposal of contaminated materials, and spill response. Participants completed the pre-survey prior to receiving the educational infographic and the post-survey immediately after reviewing it. All responses were collected anonymously. The full survey instrument is presented in the Appendices. Statistical analysis was performed by the biostatistics unit at KFMC using IBM SPSS Statistics version 29.0 (IBM Corp., Armonk, NY). Descriptive statistics were used to summarize participant demographics and survey responses. Chi-square tests were conducted to compare categorical variables between pre- and post-intervention groups. A p-value of <0.05 was considered statistically significant.

## Results

A total of 26 oral hazardous medications met the inclusion criteria for safe crushing. A summary table was generated outlining the generic and brand names, crushing instructions, and supporting references for each item (Table 1).

**Table 1:** Summary of Crushing Suitability for Oral Hazardous Medications

Drug name	Available brand	Dosage form	Preparation
Acitretin [17]	NEOTIGASON^®^	10 mg; capsule	Open the capsule and then dissolve its content with 10 mL of milk
Bosentan [18]	TRACLEER^®^	62.5 mg, 125 mg; tablet	Crush the tablet and then dissolve its content with 10 mL of water
Chlorambucil [13]	Leukeran^®^	2 mg; tablet	Crush the tablet and then dissolve its content with 10 mL of warm water
Crizotinib[13]	XALKORI^®^	250 mg, capsule	Open the capsule and then dissolve its content with 15 mL of water
Dasatinib [19]	SPRYCEL^®^	50 mg, 70 mg; tablet	Crush the tablet and then dissolve its content with 10 mL of orange juice
Enzalutamide[13]	Xtandi^®^	40 mg, capsule	Open the capsule and then dissolve its content with 20 mL of water
Everolimus[13]	AFINITOR^®^	0.25 mg, 0.75 mg, 5 mg, 10 mg; tablet	Crush the tablet and then dissolve its content with 30 mL of water
Exemestane [15]	Aromaplex^®^	25 mg; tablet	Crush the tablet and then dissolve its content with 10 mL of water
Lenalidomide [13,15]	Revlimid^®^	10 mg, 25 mg; capsule	Crush the capsule and then dissolve its content with 20 mL of warm water until complete dissolution
Lomustine [13]	Gleostine^®^	10 mg, 40 mg; capsule	Open the capsule and then dissolve its content with 20 mL of milk
Macitentan [20]	OPSUMIT^®^	10 mg; tablet	Crush the tablet and then dissolve its content with 10 mL of water
Megestrol [15,19]	Megace®	40 mg, 160 mg; tablet	Crush the tablet and then dissolve its content with 20 mL of water
Melphalan [15]	Alkeran^®^	2 mg; tablet	Crush the tablet and then dissolve its content with 10 mL of water
Misoprostol [15]	Cytotec^®^	200 mcg; tablet	Crush the tablet and then dissolve its content with 10 mL of water
Nilotinib[13]	Tasigna^®^	150 mg, 200 mg; capsule	Open the capsule and then dissolve its content with 15 mL of water
Paroxetine [15]	Seroxat^®^	20 mg; tablet	Crush the tablet and then dissolve its content with 10 mL of water
Phenoxybenzamine [14]	Dibenyline^®^	10 mg; capsule	Open the capsule and then dissolve its content with 10 mL of water
Propylthiouracil [14]	PTU^®^	50 mg; tablet	Crush the tablet and then dissolve its content with 10 mL of water
Rasagiline[21]	Ragilin^®^	1 mg; tablet	Crush the tablet and then dissolve its content with 10 mL of water
Riociguat [14]	Adempas^®^	1 mg, 1.5 mg, 2 mg, 2.5 mg; tablet	Crush the tablet and then dissolve its content with 10 mL of water
Sorafenib [13]	Nexavar^®^	200 mg; tablet	Crush the tablet and then dissolve its content with 10 mL of water
Sunitinib [13]	Sutent^®^	25 mg, capsule	Open the capsule and then dissolve its content with 10 mL of water
Tamoxifen [15]	Nolvadex-D^®^	10 mg, 20 mg; tablet	Crush the tablet and then dissolve its content with 10 mL of water
Tofacitinib [22]	Xeljanz^®^	5 mg; tablet	Crush the tablet and then dissolve its content with 10 mL of water
Tretinoin [13]	Vesanoid^®^	10 mg; capsule	Mix the required quantity with warm water until complete dissolution
Warfarin [15,23]	Coumadin^®^	1 mg, 2 mg, 2.5 mg, 5 mg; tablet	Crush the tablet and then dissolve its content with 10 mL of water

Survey result

The study included 50 participants who completed both pre- and post-intervention surveys. The majority of participants were female (56%), and the age distribution was predominantly between 20 and 40 years (68%). Educational accomplishment was relatively varied, with high school diplomas representing the most common level (36%), followed by bachelor's degrees (28%) (Table 2). Despite 67% of participants having some awareness of hazardous medications, significant gaps were identified in their understanding of associated risks (63%), as well as in their knowledge regarding proper storage and disposal practices. Only 66% indicated that they stored medications in their original packaging; conversely, 26% reported utilizing inappropriate storage locations such as bathrooms, and 7% utilized refrigerators. Disposal methods also exhibited variability; notably, 28% incorrectly stated that they washed and reused tools. Furthermore, knowledge surrounding spill management was inadequate, with only 16% expressing that they would allow spills to dry unattended (Table 3). A comparative analysis of the pre- and post-intervention groups revealed no significant demographic differences in gender, age, or education, as confirmed by Chi-square tests, thereby validating the comparability of the groups. However, all outcomes related to awareness and behavior demonstrated statistically significant improvements following the intervention (p < 0.001). Awareness of hazardous medications increased from 34% to 100%, while the recognition of associated risks rose from 26% to 100%. Furthermore, the clarity of preparation instructions improved from 40% to 100%, and proper storage practices increased from 32% to 100%. The frequency of correct disposal behavior escalated from 26% to 100%, and appropriate responses to medication spills improved dramatically from a mere 8% to 100% (Table 4).

**Table 2:** Demographic Characteristics of Participants (N = 50)

Variable	Category	Frequency (number)	Percentage (%)
Gender	Female	28	56
	Male	22	44
Age group (years)	20-40	34	68
	>40	16	32
Education level	High school	18	36
	Bachelor's degree	14	28
	Diploma	10	20
	Postgraduate	8	16

**Table 3:** Baseline Knowledge and Behavior Patterns (Selected Responses)

Parameter	Response option	Frequency (number)	Percentage (%)
Awareness of hazardous medications	Aware	33	67
Understanding of associated risks	Aware	32	63
Storage in original packaging	Yes	33	66
Inappropriate storage (bathroom)	Yes	13	26
Inappropriate storage (refrigerator)	Yes	4	7
Washed/reused tools	Yes	14	28
Allowed spills to dry unattended	Yes	8	16

**Table 4:** Pre- and Post-intervention Comparison of Knowledge and Practices Related to Hazardous Medications

Domain	Pre-intervention (number (%))	Post-intervention (number (%))	Test used	p-value	Chi-square (χ²)
Awareness of hazardous medications	17 (34%)	50 (100%)	Chi-square	<0.001	49.25
Recognition of associated risks	13 (26%)	50 (100%)	Chi-square	<0.001	58.73
Clarity of preparation instructions	20 (40%)	50 (100%)	Chi-square	<0.001	42.86
Proper storage practices	16 (32%)	50 (100%)	Chi-square	<0.001	51.52
Correct disposal behavior	13 (26%)	50 (100%)	Chi-square	<0.001	58.73
Appropriate spill management	4 (8%)	50 (100%)	Chi-square	<0.001	85.19

## Discussion

This study addresses a critical and under-recognized gap in the safe handling of oral hazardous medications (HMs), particularly in home-based settings where patients with difficult swallowing or other impairments often rely on caregivers to modify oral solid dosage forms. Despite well-documented risks including exposure to cytotoxic particulates, loss of drug stability, and altered pharmacokinetics, this practice remains common due to the lack of clear, accessible guidance [8,9]. Most hazardous medications are not designed to be crushed, and doing so can lead to degradation, changes in pH, reduced bioavailability, or therapeutic failure, while also increasing occupational exposure through aerosolization or skin contact [7]. In our study, a pharmacist-led initiative was implemented to address these challenges by providing a structured, evidence-based protocol that combined clear two-step crushing instructions, solvent recommendations, and educational materials designed to be easily understood and applied by caregivers. The initiative emphasized immediate administration after preparation, especially for drugs lacking post-manipulation stability data, particularly for agents prepared in non-sterile environments. While some data exist (e.g., warfarin), such evidence is limited and rarely provided by manufacturers [23]. Our findings also revealed that generic products were excluded from the safe-to-crush list due to the absence of validated crushing instructions in their summary of product characteristics (SPCs), consistent with concerns raised by the Royal Pharmaceutical Society about the inadequacy of guidance in both generics and several originator brands [7].

Importantly, the study showed that education plays a transformative role in ensuring safe home handling of HMs. At baseline, caregiver awareness of medication hazards was limited (26%), with only 8% able to identify appropriate spill management techniques. After participating in our pharmacist-led counseling, supported by bilingual digital infographics and informed by NIOSH (2024), BOPA (2023), and Lexicomp. Knowledge and reported handling practices improved significantly, reaching 100% accuracy across all domains (p < 0.001). These findings align with prior studies showing the effectiveness of structured education. Furthermore, our results are consistent with global data. In Sweden, a pilot study showed that structured education, including written, video, and hands-on training, improved correct technique from 19% to 89.5% (p < 0.0001) [24]. These examples reinforce the need for comprehensive, multimodal education strategies rather than reliance on verbal instructions alone. In our results, the consistently elevated post-intervention scores should be interpreted with caution. Because the assessment was conducted immediately following the educational activity, the results may reflect transient recall rather than durable knowledge acquisition. Also, the lack of extended follow-up further restricts our ability to evaluate long-term retention or the real-world application of the competencies addressed.

## Conclusions

Although our findings are promising, several limitations must be acknowledged. The study was conducted at a single center with a modest sample size, and we focused on short-term post-intervention outcomes without evaluating long-term adherence or environmental contamination. Notably, the post-intervention survey was administered immediately after the educational intervention, meaning that the uniformly high scores likely reflect short-term recall rather than sustained knowledge or behavioral change, which may overestimate long-term retention and real-world practice. Furthermore, the inclusion of the five pilot participants in the final analysis may have introduced a minor testing effect, which could slightly overestimate post-intervention performance. Additionally, access to personal protective equipment (PPE) at home remains limited, and the exclusion of generics has narrowed the range of applicable drugs. Nevertheless, the initiative suggests the potential value of pharmacist-led interventions in promoting safe drug handling practices at home. By combining practical guidance with tailored education, our approach achieved full competency in post-training assessments and offers a scalable model for wider implementation. Moving forward, regulatory bodies and pharmaceutical manufacturers should prioritize the inclusion of standardized, validated crushing instructions in product labeling, particularly for high-risk medications, to bridge the current gap between clinical safety standards and real-world patient and caregiver practices.

## Figures and Tables

**Figure 1:**
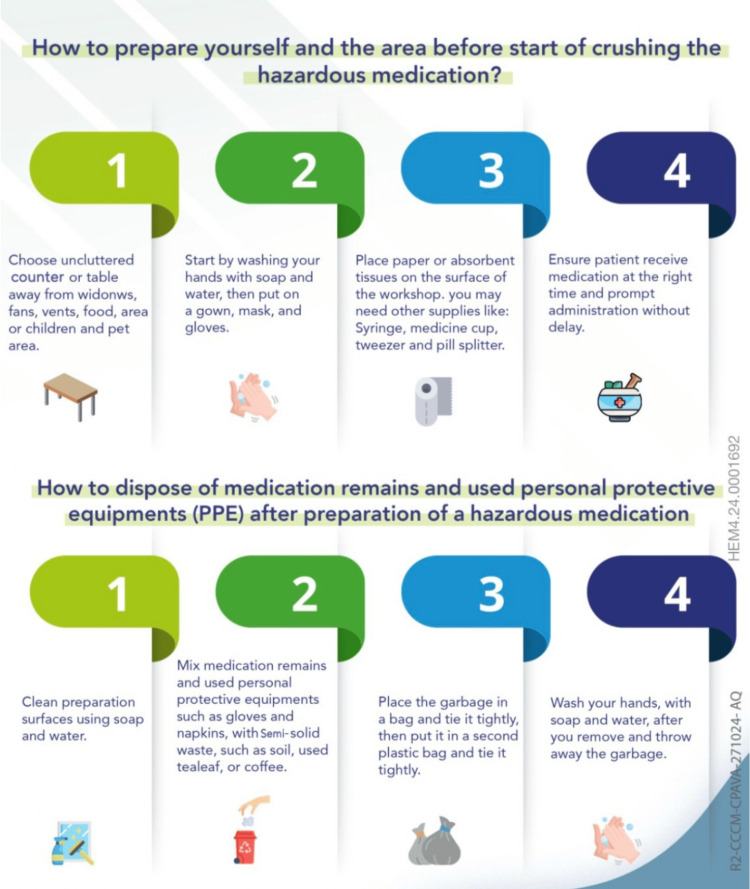
Instructional Infographic for Safe Handling of Oral Hazardous Medications Image credit: Original illustration created by the corresponding author (Faisal A. Aleidi).

**Figure 2:**
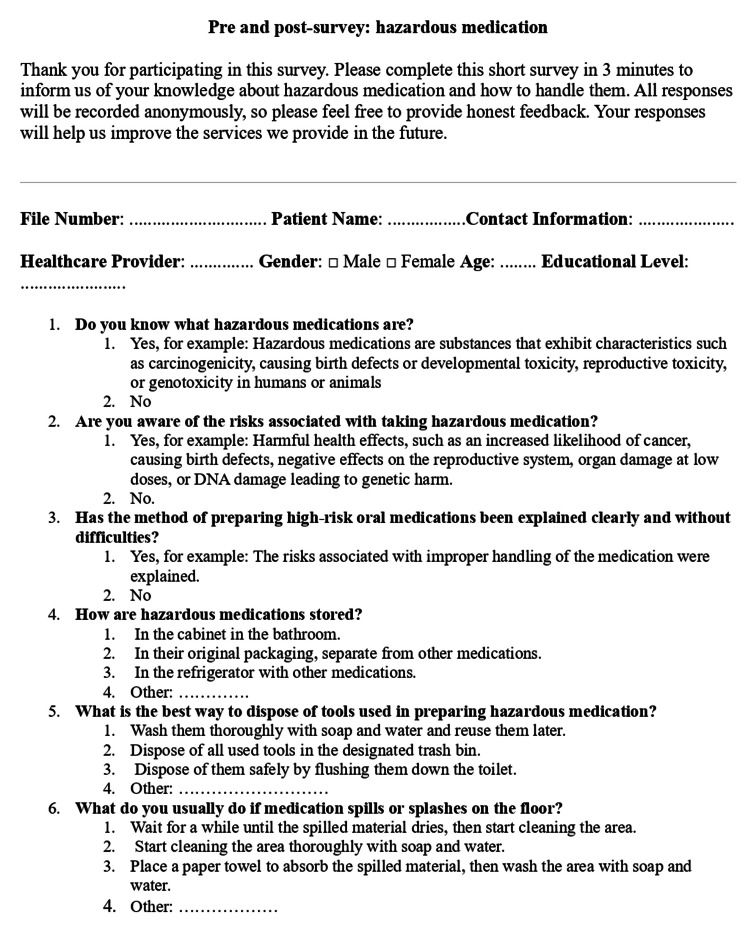
Pre- and Post-intervention Survey

## Appendices

Figure 1 presents the infographic for safe handling of oral hazardous medications.

The survey instrument used in the present study is presented in Figure 2. 
